# Determinants of hemoglobin level and time to default from Highly Active Antiretroviral Therapy (HAART) for adult clients living with HIV under treatment; a retrospective cohort study design

**DOI:** 10.1038/s41598-024-62952-w

**Published:** 2024-06-28

**Authors:** Nurye Seid Muhie, Awoke Seyoum Tegegne

**Affiliations:** 1 Department of Statistics, Mekidela Amba University, Tulu Awulia, Ethiopia; 2 Department of Bio-Statistics, Bahir Dar University, Bahir Dar, Ethiopia

**Keywords:** Level of Hgb, Time to default, Hematological parameter, Joint model, HAART, Hazard for defaulting from treatment, Biomarkers, Diseases, Health care, Medical research, Molecular medicine, Risk factors

## Abstract

HIV/AIDS is one of the most devastating infectious diseases affecting humankind all over the world and its impact goes beyond public health problems. This study was conducted to investigate the joint predictors of hemoglobin level and time to default from treatment for adult clients living with HIV/AIDS under HAART at the University of Gondar Comprehensive and Specialized Hospital, North-west Ethiopia. The study was conducted using a retrospective cohort design from the medical records of 403 randomly selected adult clients living with HIV whose follow-ups were from September 2015 to March 2022. Hemoglobin level was projected using Sahli’s acid-hematin method. Hence, the hemoglobin tube was filled with N/10 hydrochloric acid up to 2 g % marking and the graduated tube was placed in Sahli’s hemoglobin meter. The blood samples were collected using the finger-pick method, considering 22 G disposable needles. The health staff did this. From a total of 403 adult patients living with HIV/AIDS included in the current study, about 44.2% defaulted from therapy. The overall mean and median estimated survival time of adult clients under study were 44.3 and 42 months respectively. The patient’s lymphocyte count (A*HR* = 0.7498, 95% *CI*: (0.7411: 0.7587), *p*-*value* < 0.01), The weight of adult patients living with HIV/AIDS (A*HR* = 0.9741, 95% *CI*: (0.9736: 0.9747), *p*-*value* = 0.012), sex of adult clients (AHR = 0.6019, 95% *CI*: (0.5979, 0.6059), *p*-value < 0.01), WHO stages III compared to Stage I (AHR = 1.4073, 95% CI: (1.3262, 1.5078), p-value < 0.01), poor adherence level (AHR = 0.2796, 95% CI: (0.2082, 0.3705) and p-value < 0.01), bedridden patients (AHR = 1.5346, 95% CI: (1.4199, 1.6495), p-value = 0.008), and opportunistic infections (AHR = 0.2237, 95% CI: (0.0248, 0.4740), p-value = 0.004) had significant effect on both hemoglobin level and time to default from treatment. Similarly, other co-morbidity conditions, disclosure status of the HIV disease, and tobacco and alcohol addiction had a significant effect on the variables of interest. The estimate of the association parameter in the slope value of Hgb level and time default was negative, indicating that the Hgb level increased as the hazard of defaulting from treatment decreased. A patient with abnormal BMI like underweight, overweight, or obese was negatively associated with the risk of anemia (lower hemoglobin level). As a recommendation, more attention should be given to those patients with abnormal BMI, patients with other co-morbidity conditions, patients with opportunistic infections, and low lymphocytes, and bedridden and ambulatory patients. Health-related education should be given to adult clients living with HIV/AIDS to be good adherents for medical treatment.

## Introduction

Human immunodeficiency virus/Acquired immune deficiency syndrome (HIV/AIDS) is one of the most disturbing infectious diseases affecting humankind overall the world and its impact goes beyond public health problems^1^. Since, the beginning of the epidemic, about 85.6 million adults have been infected with the HIV virus and among these, about 40.4 million people have been died because of HIV related disease. Globally, about 39 million people were living with HIV at the end of 2022 and of these 630,000 were died from HIV-related causes and 1.3 million people acquired HIV^2^.

HIV/AIDS is also a leading cause of morbidity and mortality in Sub-Saharan African country^2^. Hence, about two-thirds of the world’s adult clients living with HIV/AIDS (about 23.5 million or 69%) belonged to Sub-Saharan Africa in the end of 2022^1,3^. Ethiopia, one of the Sub-Saharan African countries, has large percentage of population living with HIV/AIDS and under treatment^4^. HIV remains a crucial public health concern in the country with a prevalence rate of 0.9% ranging from 0.1 to 4.8% among adults^4^.

The Amhara region, one of the eleven regions in the country, is a pandemic area of the HIV disease where large adult clients living with the HIV/AIDS^4,5^. Even though HIV prevalence in Ethiopia decreased from year to year, the issue is still a significant epidemic burden in the region^6,7^. Hence, the region is at the 5th rank with prevalence rate of 4.1% in the country related to HIV infection among urban adults^8^.

The progression of HIV leads to a decrease in blood hemoglobin levels and is substantially associated with a decrease in the survival time of patients or waiting time patients under treatment^9^. Low hemoglobin levels are a common hematological problem among adults living with HIV. It is related to a higher risk of developing opportunistic infections^10^. The hematological parameters (hemoglobin level), and waiting time of patients under treatment can be significantly improved by early HAART initiation^11^. However, many adult clients living with the virus are defaulting from Highly Active Antiretroviral Therapy (HAART), and some are completely rejecting HAART and further leading to a substantial decrease in hematological parameters^11,12^.

Defaulting from HAART occurred because of transferring to another health centers for different reasons, defaulting because of death of patients or defaulting because of ignorance /forgotten the appointment given by the health staff. Defaulting also related to be non-medication adherence including taking pills^12^. Adult clients living with the HIV/AIDS has short waiting time under treatment if he/she defaulted from treatment and defaulter patients have decreasing survival time under treatment.

Most of the studies, conducted previously are longitudinal or survival, conducted separately^9^. Hence, a study had been conducted on blood hemoglobin measurement as a predictive indicator for the progression of HIV/AIDS in resource-limited settings^9^. Another study had been also conducted with a separate survival time to explore possible risk factors for time-to-default of adult clients living with HIV/AIDS using the Cox Proportional Hazards (Cox-PH) model^12^.

Such studies did not investigate the relationships between the two response variables namely hemoglobin level and time to default at the same time. Joint modeling of longitudinal and survival data incorporates all information about the two responses simultaneously and provides valid and efficient inferences for the given data. Hence, the joint model is very essential to overcome the problem of escaping the association between the longitudinal and survival responses. It is also important to reduce the biases of parameter estimates accounted for the association between the two responses^13^.

As far as the authors’ knowledge are concerned, there is a scarcity of studies about joint analysis of hemoglobin (Hgb) level and time to default from HAART in the study area. Therefore, the main objective of this study was to identify predictors for hemoglobin level and time to default from HAART jointly for adult clients living with HIV/AIDS under HAART at the University of Gondar comprehensive Specialized Hospital, the Amhara region, north-west Ethiopia.

## Materials and participants

### Study area and study design

This study was conducted at the University of Gondar Comprehensive Specialized Hospital Amhara region, North-west Ethiopia. The hospital belongs to the University of Gondar and it is a specialized, referral and teaching hospital. Hence, many patients from district hospitals in the catchment area are referred to this hospital and about 5000 HIV-infected patients at any ages are under treatment in this treatment center. The study area was selected because of a large number of adult clients living with HIV/AIDS registered HAART. An institutional-based retrospective cohort study design was carried out to retrieve relevant information from the medical records of adult clients living with HIV/AIDS.

### Study population, study period, and sampling procedures and inclusion criterion

The study population for this study was all medical records of adult clients living with HIV/AIDS, treated at the hospital whose follow-ups were from September 2015 to March 2022. The source of data in this study was a secondary source obtained by reviewing medical records of adult clients living with HIV/AIDS treated under the follow-up periods.

All the primary data from the respondents were collected by the health staff for treatment purpose and documented in organized manner with their own identification number within the hospital. During data collection from the patients, anthropometry was conducted using the standard protocol. Hence, height in meter was recorded without shoes by the measuring tape which was mounted on the wall. Weight in kilo grams was taken on Duchess weighing scale with light clothes and without shoes. BMI in kilograms/metre^2^ was calculated using Quetelet formula as shown below^14^:

$$ {\text{BMI }} = {\text{ Weight }}\left( {{\text{kg}}} \right)/{\text{Height }}\left( {{\text{m}}^{{2}} } \right) $$

A systematic random sampling technique was applied to select random files/charts form the documentation section. All the variables and respected values (data) from each file has been collected by the health staff. The data collection format was developed by the investigators in consultation with the health staff working in the ART section of the hospital. In collecting secondary data from the charts of patients, name of each patient was not declared in the data collection sheet rather, the identification number of patients was indicated corresponding to other variables included under study.

The study included all adult clients living with HIV/AIDS attending with a minimum of two visiting times for a longitudinal response, adult clients living with HIV/AIDS patients whose ages were at least 15 years, and patients who had started their treatment within study periods were included under this study. HIV patients with ages less than 15 years old or HIV-patients under treatment with less than two visiting times and adult clients living with HIV/AIDS with incomplete data were excluded in the current study.

### Sample size determination

The appropriate sample size determination formula used in this study was based on^14^ which is given by: $n=\frac{d}{p(defualt)}$ the total number of defaulters ($d$) can be found by using $d=\frac{4{({z}_{\frac{\alpha }{2}}+{z}_{\beta })}^{2}}{{\theta }_{R}^{2}}$, where $n$ is the required number of adult clients living to be include in this study, $\alpha$ is the level of significance or the size of the critical region in hypothesis testing and it is taken as 5% in this study, $\beta$ is the power of the test with value 80% taken for this case. From the standard normal distribution table ${z}_{\frac{\alpha }{2}}$ and ${z}_{\beta }$ the upper $\alpha /2$ and upper $\beta$ points are given as 1.96 and 0.84 respectively. Hence, the total sample size included under this study was 403.

### Quality of data

In a retrospective cohort study design, the data collection procedures were based on the patient's charts and electronic database system. In order to ensure the quality of the collected data, one-day of intensive training was given to data extractors. Pilate test was conducted on 25 randomly selected charts before a formal data collection procedures started. The necessary amendments were conducted to the final data extraction format for completeness and consistency of the data.

### Variables included in the study

#### Dependent variables

The two response variables under this investigation were hemoglobin (Hgb) level per deciliter of blood and time to default from HAART. The longitudinal biomarker (Hgb level) for adult clients living with HIV/AIDS was measured at every 6 months interval starting from baseline, 6, 12 …72 months. The survival time for each patient was also measured in months by subtracting the month of starting HAART from the month of event existence. The survival status of patients was classified as follow^10^: $$survival status=\left\{\begin{array}{c}0, if censored\\ 1, if event(defualt)\end{array}\right.$$

### Explanatory variables

The explanatory variables in the current study, selected based on the previous related literature, were weight in kg, BMI in kg/m^2^, age in years, sex, functional status, residence area, religion, marital status, education level, occupation, disclosure status, alcohol addiction, tobacco addiction, WHO Clinical stage, ART regiment, medication adherence, TB co-infection, Opportunistic infections (OIs), Other Comorbid Condition (OCC) and visiting times in a month. For further information on the categories of explanatory variables in this study, one can refer to Table 1 in the results section.

**Table 1:** Baseline socio-demographic and behavioral covariates of study participants.

Variables	Categories	Survival status		Total	Chi-square test	p-values
		Censored (%)	Event (%)			
Age	15–24 25–34 35–44 > 44	55(87.3) 47(25.82) 72(80.90) 51(73.91)	8(12.7) 135(74.18) 17(19.1) 18(26.09)	63 182 89 69	123.584	0.000
Gender	Male Female	109(76.22 ) 116(44.62)	34(23.78) 144(55.38)	143 260	37.378	0.000
Residence	Urban Rural	155 (70.45) 70(38.25)	65(29.55) 113(61.75)	220 183	42.012	0.001
Functional status	Working Ambulatory Bedridden	188(68.36) 23(22.77) 14(51.85)	87(31.64) 78 (77.2 ) 13 (48.15)	275 101 27	62.450	0.000
Marital status	Single Marriage Separated Widowed Divorced	45(53.57) 89 (48.63) 11(55.00) 30(75.00) 50 (65.79)	39(46.43) 94(51.37) 9(45.00) 10(25.00) 26(34.21)	84 183 20 40 76	13.040	0.011
Level of education	Not educated Primary Secondary Tertiary	36(66.67) 63(45.32) 72(55.38) 54(67.50)	18(33.33) 76(54.68) 58(44.62) 26(32.50)	54 139 130 80	13.222	0.004
Occupation	Merchant Housewife Daily Labor Gov't Employ Non Gov't Other	38(9.5) 61(15.1) 47(11.7) 36(8.9) 11(2.7) 34(8.4)	28(42.42) 51(45.95) 41(43.62) 3(9.09) 11(50.00) 44(57.14)	66 111 94 33 22 77	22.261	0.000
Religion	Muslim Orthodox Other	16(94.12) 192(53.04) 17(70.83)	1(5.88) 170(46.96) 7(29.17)	17 362 24	13.440	0.001
Disclosure Status	No Yes	23(33.33) 207(59.31)	36(66.67) 142(40.69)	54 349	12.799	0.000
Tobacco addiction	No Yes	180(52.17) 45(77.59)	165(47.83) 13(22.41)	345 58	13.003	0.000
Alcohol addiction	No Yes	196(63.02) 29(31.52)	115(36.98) 63(68.48)	311 92	28.569	0.000
Total		225(55.8)	178(44.2)	403		

Measures of selected variables: Hemoglobin level was computed using Sahli’s acid hematin method. Hence, the hemoglobin tube was filled with N/10 hydrochloric acid up to 2 g % marking and the graduated tube was placed in Sahli’s hemoglobin meter and the samples of blood were collected using finger pick method, considering 22 G disposable needles. Then the sample of blood was dropped in to Sahli’s tube up to 20 ML marking and the sample of blood and hydrochloric acid were mixed using stirrer. In order to change the mixture in to acid hematin, the process was conducted for about 5 min. The acid hematin was diluted using pure/distilled water with dropper until it matched with the standard color plates of comparator. The unit for results obtained from this procedure was read as g/dl and the level of hemoglobin was projected up to 0.1 g/dl. The patient was categorized as anemic if the hemoglobin level was at most 12 g/dl.

Body Mass Index (BMI) was considered as continuous variables ( any value with unit kg/m^2^) and based on Quetlet’s index these continuous values were categorized under-weight if the BMI was less than 18.5 kg/m^2^, healthy weight, if the BMI was 18.5- 25 kg/m^2^, overweight, if the BMI was 25–30 kg/m^2^ and obesity, if the BMI was at least 30 kg/m^2.^. Correlation between hemoglobin concentration and BMI was assessed using Pearson’s correlation coefficient.

### The generalized linear mixed effects model

In longitudinal data analysis, the outcome variable was measured repeatedly over time for each of the participants/subjects. In this case, there were two sources of variations: within and between subject variations. Modeling within patient variation allows us to study changes over time while modeling between-patient variation allows understand changes between patients/subjects^14^. Random effects were used to generate correlation structures between repeated measurements in longitudinal studies^14–16^. The linear mixed effect model was used to model the repeatedly measured longitudinal data that quantifies the relationships between a continuous dependent variable and various covariates^17^.

Let Y_ij_ (i = 1,2,…,n; j = 1,2,…, n_i_) be the hemoglobin level of patient i at follow up visit time j; Y_i_ = ${( {y}_{i1} , {y}_{i2} , \dots ..., {y}_{ini} )}^{T}$ a random vector; ${{\varvec{X}}}_{i1}^{T}$ be an n_i_ × p design matrix of fixed effects for patient i that is associated with the p-dimensional vector β_i_ of fixed effects; and ${Z}_{i1}^{T}$ be an n_i_ × q design matrix of random effects for patient i that is associated with the q dimensional vector ν_i_ of patient specific random effects. Then, if the conditional distribution of Yi given ν_i_ is from the exponential family, the generalized linear mixed effects model for Yi can be written as^17^:

$$\text{g}\left({Y}_{i}\right)= {X}_{i1}^{T}\beta \text{i}+{Z}_{i1}^{T}\nu \text{i}+ {\varepsilon }_{i}$$

where g(.) is the link function that is completely specified by the conditional of y_i_ in the exponential family. Since, the response of the current investigation was continuous, the unit link function was used in this study.

Semi-parametric survival model: One of the most popular types of regression models in the case of semi-parametric is the Cox proportional hazards (Cox PH) regression model^15^. The Cox PH model consists of two parts; the hazard function often denoted by describing how the hazard (risk) changes over time at baseline levels of covariate and the covariate with exponential of product with their coefficients^15^. The Cox PH semi-parametric model has a common choice for the time-to-event model.

The hazard function is a measure of the probability of failure during a very small interval, assuming that the individual has survived at the beginning of the interval^16^. The hazard function describes the concept of the risk of an outcome (defaulter) in an interval after time t, conditional on the subject having survived to time t^16^.

Method of data analysis: R software of version 4.3 was used for data analysis. In data analysis, exploratory data analysis, individual profiles plot, and exploring mean structure were conducted. Similarly, the covariance structures were compared and the one with the smallest AIC and BIC were selected. Hence, among the different covariance structures, un-structure was selected for the current study. Missing data management was also conducted using multiple data imputation techniques.

### Ethics approval and consent to participate

All methods were performed in accordance with the ethical standards as laid down in the Declaration of Helsinki and its later amendments or comparable ethical standards. Informed consent was obtained from all the participants or their legal guardian for the study. Hence, the experimental protocol was approved by the Bahir Dar University licensing committee.

## Results

The socio-demographic, behavioral, and clinical characteristics of baseline categorical variables together with the observed survival process are described in Tables 1 and 2. Out of 403 study subjects/participants, 64.5% were female and 35.5% were male adult clients living with HIV/AIDS. The majority of the participants in the current study were at working functional status (68.2%) and orthodox religious followers (89.8%), and around half of the patients (45.2%) were between the age group 25–34 years (refer to Table 1). Regarding adherence status, about 56.3% of poor adherents, 50.6% of fair adherents, and 41.7% of good adherents have been defaulted from HAART (refer to Table 2).

**Table 2:** Baseline clinical variables of participants.

Variables	Categories	Survival Status		Total	Chi-square test	p-values
		Censored (%)	Event (%)			
Adherence	Poor Fair Good	7(43.75) 43(49.43) 175(58.33)	9(56.25) 44(50.57) 125(41.67)	16 87 300	186.875	0.000
WHO clinical stage	Stage-I Stage-II Stage-III Stage-IV	114(50.89) 53 (68.83) 47(63.51) 11 (39.29)	110(49.11) 24(31.17) 27(36.49) 17 (60.71)	224 77 74 28	12.372	0.006
ART regiment	1c(AZT-3TC-NVP) 1d(AZT-3TC-EFV) 1e(TDF-3TC-EFV) 1f.(TDF + 3TC + NVP) 1j(TDF + 3TC + DTG) Other	42(61.76) 39(88.64) 78(40.84) 26(81.25) 13(48.15) 27 (65.85)	26(38.24) 5(11.36) 113(59.16) 6(18.75) 14 (51.85) 14(34.15)	68 44 191 32 27 41	48.285	0.000
TB screen	No Yes	216(62.25) 9(16.07)	131(37.75) 47(83.93)	347 56	41.693	0.000
OIs	No Yes	198(61.68) 27( 32.93)	123(38.32) 55(67.07)	321 82	21.901	0.000
OCC	No Yes	203(64.65) 22 (24.72)	111(35.35) 67(75.28 )	314 89	44.837	0.001
Total		225(55.8)	178(44.2)	403		

The baseline continuous characteristics of participants included in this study are indicated in Table 3. Table 3 indicates that the mean hematocrit of 403 participants under study was 37.7% with its corresponding standard deviation of 4.50% and with minimum and maximum hematocrit of 20.1% and 54.3% respectively. The mean weight of patients was 46.94 kg. with a corresponding standard deviation of 10.9 kg and the mean of WBC, RBC, and Platelet cell count of adult clients living with HIV/AIDS with their corresponding standard deviation were 6.01 (1.88)10^^3^/$\mu l$, 4.17 (1.35), 10^^6^/$\mu l$, and 286.93 (95.25) 10^^3^/$\mu l$ respectively(Reference to Table 3).

**Table 3:** Baseline socio-demographic and clinical continuous predictors.

Variables	Minimum	Maximum	Mean	Std. deviation
Hematocrit in %	20.80	54.30	37.66	4.50
Weight in kg	33.0	85.00	46.94	10.9
Body mass index in kg/m^2^	11.13	34.05	18.49	4.00
White blood cell in 10^^3^/$\mu l$	1.60	13.00	6.01	1.88
Red blood cell in 10^^6^/$\mu l$	2.00	11.00	4.17	1.35
Platelet in 10^^3^/$\mu l$	23	641	286.93	95.25
Lymphocyte in %	15.20	69.10	42.69	11.32
Monocytes in %	2.0	13.4	7.33	3.13

Std. deviation indicates standard deviation of continuous predictors.

Longitudinal linear mixed effect models: The mean hemoglobin level of adult clients living with HIV/AIDS under HAART at baseline was 12.87 g/dl with a corresponding standard deviation of 3.0392 g/dl. Smaller mean Hgb levels of adult clients living with HIV/AIDS were observed at the baseline period and increased continuously as visiting time increased (72 months).

Before proceeding to the longitudinal data model building, the possible covariates were selected for the multivariable linear mixed model at a 25% level of significance in the univariate data analysis.

Model comparisons of linear mixed-effects model: Different candidate models with different random effects were considered for longitudinal measures of the Hgb level of the adult clients living with HIV/AIDS. The most appropriate random effect model for the longitudinal measures was selected using the deviance information criteria (DIC). Hence, random intercepts and random slopes for the Hgb level under the full model were selected because of the smaller value of DIC.

Joint model results: Joint model of the two response variables was conducted considering different latent processes for the repetitive measures of hemoglobin level. The joint model was conducted assuming that there are homogeneous and heterogeneous variances for the variable of interest. Hence, the longitudinal sub-model was described using conventional generalized linear mixed effects model including patient-explicit variances. On the other hand, the time to default from the HAART sub-model was fitted using a Cox proportional hazard (Cox PH) model and the two sub-models were correlated considering common covariates. The joint model of the two response variables is indicated in Table 4. For the table to be manageable within one page, all rows with insignificant variables are deleted in this joint data analysis.

**Table 4:** Joint model parameter estimates for HGB and time-to-event sub-models.

Variables	Post mean	Std. error	HR	95% CI for HR		p-value
				Lower	Upper	
Hematocrit	0.0052	0.0003	1.0052	0.9862	1.0255	0.552
Weight	0.0262	0.0003	0.9741	0.9736	0.9747	0.012
BMI	0.0896	0.0008	0.9143	0.8713	0.9615	0.000
Platelet	0.0757	0.0230	0.9271	0.8862	0.9698	0.001
Lymphocyte	0.2879	0.0006	0.7498	0.7411	0.7587	0.000
Sex	0					
Female	0.5077	0.0034	0.6019	0.5979	0.6059	0.000
Age in year (< 25 years)	0					
25–34 35–44 > 44	1.8794 1.1391 1.3831	0.0084 0.0065 0.0075	6.5496 3.1239 3.9872	4.4119 1.9943 2.5196	10.1017 4.8666 6.3414	0.000 0.000 0.000
Adherence(Ref. = poor)	0					
Fair Good	0.2719 1.2742	0.0028 0.0044	0.7619 0.2796	0.6313 0.2082	0.9036 0.3705	0.006 0.000
WHO stage(ref. = Stage I)	0					
Stage-II Stage-III Stage-IV	0.5766 0.8982 0.3159	0.0035 0.0035 0.0048	0.5618 0.4073 0.7291	0.4474 0.3261 0.5289	0.7195 0.5078 0.9681	0.000 0.000 0.036
Working status	0					
Ambulatory Bedridden	− 0.0291 − 0.4283	0.0028 0.0049	1.0295 1.5346	1.0064 1.4199	1.2433 1.6495	0.004 0.008
TB screen (Ref. = yes)	0					
No	0.5618	0.0029	0.7538	0.4696	0.9951	0.000
OIs (Ref = yes)	0					
No	0.2019	0.0028	0.2237	0.0248	0.4740	0.024
OCC (Ref. = yes)	0					
No	0.2675	0.0020	0.3367	0.3016	0.4118	0.004
Disclosure (Ref. = no)	0					
Yes	0.4077	0.0030	0.6652	0.5475	0.7939	0.000
Tobacco addiction (ref = no)	0					
Yes	− 0.7472	0.0047	2.1111	2.0917	2.1306	0.000
Alcohol addiction (ref = no)	0					
Yes	− 0.4410	0.0023	1.5543	1.2947	1.8965	0.000

Table 4 indicates that the mean fixed effect intercept estimated coefficient in the longitudinal sub-model was 11.38 g/dl, given that the other covariates are constant. The result indicates that visiting time was a significant covariate for longitudinal responses. Hence as visiting time increased by one month, the expected hemoglobin level was increased by 0.0475 g/dl, and the hazard of defaulting patients from treatment was decreased by 32.5%, holding all other covariates constant (p-value = 0.002).

As the body mass index of a patient increased by one kg/m^2^ (for low BMI patients) the expected hemoglobin level of the patients was increased by 0.0896 and the corresponding hazard of defaulting from the treatment was decreased by 8.4%, given the other covariates constant (p-value < 0.01). Compared low BMI patients with healthy BMI, the expected hemoglobin level of low BMI was 0.0896 times that of healthy BMI given the other covariates constant (p-value < 0.01). Similarly, comparing high BMI with health BMI, the expected level of hemoglobin for high BMI was 0.076 times that of healthy BMI (p-value < 0.01). Hence, the current study indicates that abnormal BMI (very low and very high BMI) had negative correlation with hemoglobin level.

The patient’s Platelet cell count had a significant effect on both Hgb level and time to default. As the patient’s platelet cell count increased by a 10^^3^/ml, the expected Hgb level was increased by 0.0757 g/dl and the corresponding hazard of defaulting from treatment was decreased by 7.3%, holding the other covariates constant (*p*-*value* = 0.001).

The patient’s lymphocyte count had a significant effect on both Hgb level and time to default. As the lymphocyte count of a patient increased by one unit, the expected Hgb was increased by 0.2879 **g/dl**, and the corresponding hazard of defaulting from the treatment was decreased by 25.1% holding for other covariates constant (A*HR* = 0.7498, 95% *CI*: (0.7411: 0.7587), *p*-*value* < 0.01).

The weight of adult clients living with HIV had a significant effect on their Hgb level and time to default. As the patient’s weight increased by one kg, the expected level of Hgb was increased by 0.0262 g/dl and the corresponding hazard of defaulting from the treatment was decreased by 2.6%, given the other covariates constant (A*HR* = 0.9741, 95% *CI*: (0.9736: 0.9747), *p*-*value* = 0.012).

Comparing female with male patients, the expected level of Hgb of females was increased by 0.5077 g/dl and the corresponding hazard of defaulting from the treatment was decreased by 40%, given the other covariates constant (AHR = 0.6019, 95% *CI*: (0.5979: 0.6059), *p*-value < 0.01).

The result in Table 4 also indicates that WHO stages had a significant effect on the variables of interest. Hence, comparing WHO stage II with WHO stage I, the expected Hgb level of WHO stage II was decreased by 0.5766 as compared to WHO stage I, and the corresponding hazard of defaulted from the treatment for WHO stage II patients was increased by 56%, given the other covariates constant (AHR = 1.5618, 95% CI: (1.4474, 1.7195), p-value < 0.01). Similarly, comparing the WHO stage III with WHO stage I, the expected level of Hgb for WHO stage III was decreased by 0.8982 g/dl, and the corresponding hazard of defaulted from the treatment by WHO stage III patients was increased by 40.7% given the other conditions constant (AHR = 1.4073, 95% CI: (1.3262, 1.5078), p-value < 0.01).

Adherence had a significant effect on the variables of interest. Comparing fair adherent patients with poor adherent patients, the expected level of fair adherent patients was increased by 0.2719 g/dl and the corresponding hazard of defaulting from the treatment was decreased by 24.8%, given the other conditions constant (AHR = 0.7619, 95% CI: (0.6313, 0.9036), p-value = 0.006). In the same way, comparing good adherent patients with poor adherent patients, the expected level of Hgb for good adherent patients was 1.2742 g/dl times that of poor adherent patients, and the corresponding hazard of defaulting from the treatment was decreased by 72.1% as compared to that of poor adherent patients, given the other conditions constant (AHR = 0.2796, 95% CI: (0.2082, 0.3705) and p-value < 0.01).

HIV functional status was another variable that significantly affected the variable of interest. Hence, the comparing ambulatory patients with patients at working status, the expected level of Hgb for ambulatory adult clients living with HIV/AIDS was decreased by 0.0291 g/dl and the corresponding hazard of defaulting from treatment was increased by 2.9% (AHR = 1.0295, 95% CI: (1.0064, 1.2433), p-value = 0.004). Similarly, the expected level of Hgb for bedridden adult clients living with HIV/AIDS was decreased by 0.4283 g/dl as compared to patients with working status, and the corresponding hazard of defaulting from the treatment was increased by 53%, given the other conditions constant (AHR = 1.5346, 95% CI: (1.4199, 1.6495), p-value = 0.008).

Opportunistic infections significantly affected the variables of interest. Hence, comparing those adult clients living with HIV/AIDS who had opportunistic infections with those without infections, the expected level of Hgb for patients without opportunistic infection was increased by 0.2019 g/dl, and the corresponding hazard of defaulting from treatment was decreased by 97.5% as compared to adult client living with HIV/AIDS who had opportunistic infections, given the other conditions constant (AHR = 0.2237, 95% CI: (0.0248, 0.4740), p-value = 0.004).

Similarly, the variables such as other co-morbidity conditions, disclosure status of the HIV disease, tobacco and alcohol addiction had a significant effect on the variables of interest.

## Discussions

The association parameter estimated at the joint model analysis was considerably different from zero and this is an indication of the correlation between the two sub-models. The estimate of the association parameter due to the slope of Hgb was negative. This means that the slope of Hgb is negatively associated with the hazard of default. This finding was consistent with one of the previously conducted studies^15,16^.

Visiting time of patients was positively associated with the expected value of Hgb level and negatively associated with the hazard to default from the treatment. Hence, as the visiting time of a patient increased the corresponding expected value of Hgb level increased and the hazard of defaulting from treatment decreased. The potential reason for this might be the fact that proper follow-ups lead to the progression of Hgb and this further encourages the patients strictly follow his/her treatment. This result was in line with the previous studies^15,17^.

Hematocrit has a great association with the level of hemoglobin and the time to default from treatment. This result indicates that a higher value of hematocrit leads to a higher level of homogony. However, those patients with higher hematocrit had a lower risk of defaulting from treatment. This finding is in line with study conducted in Nigeria^18^.

The result of this study showed that the body mass index (BMI) of patients was a significant predictor for the response, Hgb level. The result in the current study indicates that a patient with Abnormal BMI like underweight (low BMI), overweight or obese, were associated with the risk of anemia and there was a negative association between hemoglobin concentration and abnormal BMI among patients under treatment. This result is consistent with one of the previous study^18^. This result was also consistent with the study conducted by^15^. The result of this study implied that the hazard of defaulters decreases with the increasing BMI for patients with low BMI but the hazard of defaulters increase with decreasing of BMI for patients with very high BMI. This result is supported by another study^19^.

The result obtained in this study also indicates that the platelet cell count was a statistically significant factor for the longitudinal Hgb level of adult clients living with HIV/AIDS. The result indicates that the expected Hgb level increases with the increasing rate of platelet cell count of patients. This study is consistent with other studies^20,21^.

HIV disease functional status of patients had statistically significant effects on hemoglobin level of patients under treatment. Hence, adult clients living with HIV/AIDS under bedridden functional status have lower expected level of Hgb as compared to working status. This study is consistent with another study^22^. Regarding the hazard of defaulting from treatment of adult clients living with HIV/AIDS, the result indicates that bedridden adult clients living with HIV/AIDS have a higher risk of defaulting as compared to that of working status. This result is consistent with the studies conducted in South-west Ethiopia^23,24^.

The result in current study also indicates that the weight of adult clients living with HIV/AIDS had positive correlation with the expected level of Hemoglobin level. Hence, the expected level of Hgb increased with the increasing rate of weight of patients. On the other hand, weight of patients and the hazard of defaulting from treatment for adult clients living with HIV/AIDS were negatively associated. This result is supported by the previous study^24^.

ART adherence was another factor significantly associated with Hgb level and the hazard of defaulting from the treatment. Good ART adherent patients have higher expected level of Hgb as compared to poor adherent patients. The result of this study indicates that good and fair adherent HIV-positive patients had a lower risk of defaulting from HAART as compared to poor adherent patients. This finding is consistence with other studies^25,26^.

This study also indicates that WHO clinical stage-III and IV adult HIV-positive patients have lower Hgb than patients with WHO clinical stage-I patients. This result was supported by other studies^27,28^. This result is also consistent with a study done by^29^. Similarly, the WHO clinical stage of adult HIV-positive patients was a significant risk factor for the hazard of defaulting from ART treatment. This study was consistent with another study^30^.

Regarding tobacco addiction, the Hgb level of tobacco-addicted adult HIV-positive patients was significantly lower in number as compared to non-addicted patients. This result is in line with other previously conducted studies done at Arba Minch, Ethiopia^17,31^. The result in the current study also indicates that the hazard of defaulting from treatment for tobacco addicted patients was higher as compared to non-addicted patients. This result is consistent with a study conducted by Deribe et al.^32^.

The patient with alcohol addiction has a lower level of Hgb. This result is contradicted by the study done in Ethiopia^33,34^. In this study, alcohol-addicted patients have a lower expected level of Hgb than non-addicted patients. The risk of defaulting from treatment is higher among alcohol-addicted patients as compared with non- alcohol addicted patients. The result of this study is consistent with a study conducted by^35^. Similarly, patients with a higher lymphocyte count have higher Hgb levels and consistent with another study conducted by^36^.

Sex has a significant association with the variables of interests namely Hgb level and the risk of defaulting from treatment. Female adult clients living with HIV/AIDS have higher Hgb levels and a lower risk of defaulting from the treatment. The result of this study was consistent with another study conducted by^37^.

The age of patients has a great role for the variation of level of Hgb and risk of defaulting from the treatment. This result is inconsistence with a study conducted in Ethiopia^38^. Under this study, patients’ history of OIs had a significant association with their default time, where patients who had a history of OIs had a higher risk of defaulting as compared to patients without history OIs.

## Conclusion

In conclusion, the results of both separate and joint model analyses were conducted in this study. The coefficient of association parameter was different from zero and statistically significant. The two responses were negatively correlated in such a way that an increase in Hgb leads to a decrease in the hazard of defaulting from the treatment. Among the predictor variables, BMI was significant for the longitudinal outcome variables namely hemoglobin level. Hence, a patient with Abnormal BMI like underweight, overweight or obese, were associated with the risk of anemia and there was a negative association between hemoglobin concentration and abnormal BMI among patients under treatment.

Among the covariates, visiting time, hematocrit, BMI, platelet cell count, lymphocyte count, sex, WHO clinical stage, functional status, OIs, tobacco addiction, alcohol addiction, disclosure status of the disease and adherence level of patients significantly affected both Hgb level and hazard of defaulting from the treatment.

AS a recommendation, the authors in current study recommended that health experts should conduct health-related education for patients to be good adherent for the prescribed medication and strict followers of their treatment.

### Limitation of the study

This research is not without limitations. One of the limitations is that some variables such as nutritional status, income status, homeownership, viral load status, and other hematological parameters like Eosinophil, Neutrophil, and Basophil counts were not available on the patient’s chart that might be associated with the Hgb level and risk of defaulting from the treatment. Having such additional variables may have additional information related to Hgb level and risk of defaulting from treatment. Hence, this study recommended future investigations including such variables. The research was conducted in a single center and this may be another limitation for the current investigation. Having additional centers may increase the information from the current study that may add value for policy implication. Hence, the authors recommend conducting further researches including other treatment centers for the current result to be more valid and consistent or to get additional information.

## Data Availability

The datasets used in the current study available from the corresponding author can be presented on reasonable request.

## References

[1] Weiss R (2018). Special anniversary review: Twenty-five years of human immunodeficiency virus research: Successes and challenges. Clin. Exp. Immunol..

[2] Moodley J (2011). Strengthening HIV services for pregnant women: An opportunity to reduce maternal mortality rates in Southern Africa/sub-Saharan Africa. BJOG Int. J. Obstetr. Gynaecol..

[3] Amuche, N. J., Emmanuel, E. I., & Innocent, N. E. HIV/AIDS in sub-Saharan Africa: current status, challenges and prospects (2017).

[4] Girum T (2018). Gender disparity in epidemiological trend of HIV/AIDS infection and treatment in Ethiopia. Arch. Public Health.

[5] Worku ED, Asemahagn MA, Endalifer ML (2020). Epidemiology of HIV infection in the Amhara region of Ethiopia, 2015 to 2018 surveillance data analysis. HIV/AIDS (Auckland, NZ).

[6] Wario KG, Tiki TG (2020). Prevalence of HIV/AIDS in West Hararghe Zone, Oromia Regional State, Ethiopia from 2016 2019. SINET Ethiopian J. Sci..

[7] Worku WZ (2022). HIV is still a major public health problem among pregnant women attending ANC in Referral Hospitals of the Amhara Regional State, Ethiopia: A cross sectional study. BMC Womens Health.

[8] Tegegne AS (2021). Predictors associated with the variation of CD4 cell count and Body Mass Index (BMI) for HIV positive adults under ART. Sci. Afr..

[9] Obirikorang C, Yeboah FA (2016). Blood haemoglobin measurement as a predictive indicator for the progression of HIV/AIDS in resource-limited setting. J. Biomed. Sci..

[10] Manaye, Y., Asrat, A., & Mengesha, E.W. Time to development of anemia and predictors among HIV-infected patients initiating ART at Felege Hiwot referral Hospital, Northwest Ethiopia: A retrospective follow-up study. *BioMed Res. Int.* **2020** (2020). 10.1155/2020/7901241 PMC7085871 32258143

[11] Damtie S (2021). Hematological abnormalities of adult HIV-infected patients before and after initiation of highly active antiretroviral treatment at debre tabor comprehensive specialized hospital, northcentral ethiopia: a cross-sectional study. HIV/AIDS (Auckland, NZ).

[12] Sylvère, T., Default Time from Tuberculosis Treatment in the Southern Republic of Benin Using Mixture Cure Model for Survival Analysis. Biom Biostat Int J 2 (5): 00039. 10.15406/bbij. 2015.02. 00039 varied geographically [8]. The annual risk of infection was 0.8% for 2008 and TB specifically was responsible for 18 deaths per 100000 populations per year [6]. The yearly death rate ranged from 6% to 14% with a decreasing trend since 2002. HIV/AIDS is an opportunist disease that TB patients have to face. *Benin, the rate of co-infection with HIV/AIDS has fluctuated between*. **13** (2015).

[13] Guo X, Carlin BP (2004). Separate and joint modeling of longitudinal and event time data using standard computer packages. Am. Stat..

[14] Ma Y, Mazumdar M, Memtsoudis SG (2012). Beyond repeated-measures analysis of variance: Advanced statistical methods for the analysis of longitudinal data in anesthesia research. Reg. Anesth. Pain Med..

[15] Seid, N. Analysis of predictors for Cd4 cell count and hemoglobin level with time to default jointly for hive positive adults under treatment at university of gondar comprehensive specialized hospital; Gondar, Ethiopia (2022). 10.1186/s13104-023-06625-3 PMC10693704 38042846

[16] Tsenkova VK (2021). Socioeconomic status and psychological well-being predict cross-time change in glycosylated hemoglobin in older women without diabetes. Psychosomatic Med..

[17] Belete Anjullo, B., & Asfaw Teni, D. Linear mixed modeling of CD4 cell counts of HIV-infected children treated with antiretroviral therapy (2021)

[18] Zheng F (2002). Factors predicting hospital stay, operative time, blood loss, and transfusion in patients undergoing revision posterior lumbar spine decompression, fusion, and segmental instrumentation. Spine.

[19] Sabasaba A (2019). Effect of isoniazid preventive therapy on tuberculosis incidence and associated risk factors among HIV infected adults in Tanzania: A retrospective cohort study. BMC Infect. Dis..

[20] Kahler CW (2017). Direct and indirect effects of heavy alcohol use on clinical outcomes in a longitudinal study of HIV patients on ART. AIDS Behav..

[21] Gebremedhin, K.B., & Haye, T.B. Factors associated with anemia among people living with HIV/AIDS taking ART in Ethiopia. Adv. Hematol. **2019** (2019). 10.1155/2019/9614205 PMC6421011 30941180

[22] Tesfamariam K, Baraki N, Kedir H (2016). Pre-ART nutritional status and its association with mortality in adult patients enrolled on ART at Fiche Hospital in North Shoa, Oromia region, Ethiopia: A retrospective cohort study. BMC Res. Notes.

[23] Odafe S (2012). Patients’ demographic and clinical characteristics and level of care associated with lost to follow-up and mortality in adult patients on first-line ART in Nigerian hospitals. J. Int. AIDS Soc..

[24] Amberbir A (2022). Predictors of adherence to antiretroviral therapy among HIV-infected persons: A prospective study in Southwest Ethiopia. BMC Public Health.

[25] Biset Ayalew, M., Mortality and its predictors among HIV infected patients taking antiretroviral treatment in Ethiopia: a systematic review. *AIDS Res. Treat.* **2017** (2017) 10.1155/2017/5415298 PMC5682904 29214077

[26] Tegegne TK (2018). The impact of geographic access on institutional delivery care use in low and middle-income countries: Systematic review and meta-analysis. PloS One.

[27] Damtew, B., Mengistie, B., & Alemayehu, T. Survival and determinants of mortality in adult HIV/Aids patients initiating antiretroviral therapy in Somali Region, Eastern Ethiopia. Pan Afr. Med. J. **22**(1) (2015). 10.11604/pamj.2015.22.138.4352 PMC4742016 26889319

[28] Nabizadeh F (2022). Autologous hematopoietic stem-cell transplantation in multiple sclerosis: A systematic review and meta-analysis. Neurol. Therapy.

[29] Alaee, E.Q., et al., The efficacy of transdiagnostic cognitive behavioural therapy on reducing negative affect, anxiety sensitivity and improving perceived control in children with emotional disorders-a randomized controlled trial. *Res. Psychother. Psychopathol. Process Outcome* **25**(1) (2022). 10.4081/ripppo.2022.588 PMC9153761 35532025

[30] Seyoum D (2017). Risk factors for mortality among adult HIV/AIDS patients following antiretroviral therapy in Southwestern Ethiopia: An assessment through survival models. Int. J. Environ. Res. Public Health.

[31] Dalbo M, Tamiso A (2016). Incidence and predictors of tuberculosis among HIV/AIDS infected patients: A five-year retrospective follow-up study. Adv. Infect. Dis..

[32] De Socio GV (2020). Is it feasible to impact on smoking habits in HIV-Infected patients? Mission impossible from the STOPSHIV Project cohort. JAIDS J. Acquir. Immune Defic. Syndr..

[33] Thoma E (2015). Changes of some blood count variables in correlation with the time of alcohol abuse. J. Addict. Res. Ther..

[34] Alzahrani T (2018). Association between electronic cigarette use and myocardial infarction. Am. J. Prevent. Med..

[35] Muture BN (2011). Factors associated with default from treatment among tuberculosis patients in Nairobi province, Kenya: A case control study. BMC Public Health.

[36] Azab B (2013). Pretreatment neutrophil/lymphocyte ratio is superior to platelet/lymphocyte ratio as a predictor of long-term mortality in breast cancer patients. Med. Oncol..

[37] Takarinda KC (2015). Gender-related differences in outcomes and attrition on antiretroviral treatment among an HIV-infected patient cohort in Zimbabwe. Int. J. Infect. Dis..

[38] Froessler B (2016). The important role for intravenous iron in perioperative patient blood management in major abdominal surgery: A randomized controlled trial. Ann. Surg..

